# Cytoplasmic Dynamics of the General Nuclear Import Machinery in Apically Growing Syncytial Cells

**DOI:** 10.1371/journal.pone.0085076

**Published:** 2013-12-20

**Authors:** Oier Etxebeste, María Villarino, Ane Markina-Iñarrairaegui, Lidia Araújo-Bazán, Eduardo A. Espeso

**Affiliations:** 1 Department of Cellular and Molecular Biology, Centro de Investigaciones Biológicas, Consejo Superior de Investigaciones Científicas, Madrid, Spain; 2 Department of Applied Chemistry, Faculty of Chemistry, University of the Basque Country, San Sebastian, Spain; 3 Department of Molecular Microbiology and Infection Biology, Centro de Investigaciones Biológicas, Consejo Superior de Investigaciones Científicas, Madrid, Spain; Universidade de Sao Paulo, Brazil

## Abstract

Karyopherins are transporters involved in the bidirectional, selective and active transport of macromolecules through nuclear pores. Importin-β1 is the paradigm of karyopherins and, together with its cargo-adapter importin-α, mediates the general nuclear import pathway. Here we show the existence of different cellular pools of both importin-α and -β1 homologues, KapA and KapB, in the coenocytic ascomycete *Aspergillus nidulans*. Fluorescence analysis of haploid and diploid strains expressing KapB::GFP and/or KapA::mRFP showed patches of both karyopherins concurrently translocating long distances in apically-growing cells. Anterograde and retrograde movements allowed those patches to reach cell tips and distal regions with an average speed in the range of μm/s. This bidirectional traffic required microtubules as well as kinesin and dynein motors, since it is blocked by benomyl and also by the inactivation of the dynein/dynactin complex through *nudA1* or *nudK317* mutations. Deletion of Kinesin-3 motor UncA, required for the transport through detyrosinated microtubules, strongly inhibited KapA and KapB movement along hyphae. Overall, this is the first report describing the bidirectional dynamics of the main nuclear import system in coenocytic fungi. A functional link is proposed between two key cellular machines of the filamentous fungal cell: nuclear transport and the tip-growth apparatus.

## Introduction

Cells develop polarity to orient their activities in a variety of different ways [1]. For example, neurons are highly polarized, with clearly segregated dendritic and axonal domains [2,3]. On the contrary, round cells such as those from budding yeast display only polarized growth during certain phases of their life cycle [4]. Establishment and maintenance of polarity within a cell requires crucial events such as the correct recruitment of the machinery involved and appropriate vesicle traffic via the cytoskeleton [1,5,6].

Polarized growth is continuous and indefinite in vegetative hyphae of filamentous fungi, such as the model ascomycetes *Neurospora crassa* and *Aspergillus nidulans* [7]. Vegetative hyphae are non-specialized, pluripotent cells that extend apically by the addition of new material to the cell wall at the tip [8]. Tight coordination between actin and tubulin cytoskeletons (and the corresponding molecular motors) is crucial for the delivery of wall materials [9] and thus the maintenance of hyphal tip extension (see for example 10–12). Building components are distributed to the tip by an apical body called *Spitzenkörper* [13] utilizing myosin motors and actin filaments [14,15]. New cell-wall components are initially contained within vesicles or endosomes that are transported from distal regions of hyphae to the apical body [5,16]. This occurs on microtubules (MT), long filaments that are nucleated from MT-organizing centers (MTOC). MTs are rather stable at the minus end and exhibit alternating rounds of growth and shrinkage at the plus end [17,18]. Molecular cargoes are transported by kinesins and dynein along microtubules [7]. The cooperation of both motors mediates endosome movement, and thus cargo transport, over the length of the entire fungal cell [19].

Nuclei were the first MT-dependent cargo described in filamentous fungi [20,21] and both MT and actin filament networks are utilized in related processes such as nuclear transport [22]. This is the selective translocation of macromolecules between the nucleus and the cytoplasm, and occurs actively through the nuclear pore complex (NPC; [23,24]). NPCs are embedded in the nuclear envelope (NE) and are composed of more than 30 different proteins. Those proteins called nucleoporins or Nup-s [24,25] have special significance in the structure and function of NPCs. The shuttle of macromolecular substrates through the NPC is dynamically mediated by a family of proteins called karyopherins [26], primarily importin-β1 followed by other members of the karyopherin-β family (see below; [27]). Twenty-two karyopherins have been identified in mammals and 15 in *Saccharomyces cerevisiae* [28,29]. Recently, the function and cellular distribution of the 14 *A. nidulans* karyopherins has been systematically characterized [26,30].

Karyopherins can bind substrates directly or via adaptors, but the targeting of the substrate into or out of the nucleus is determined by the presence in its amino acidic chain of a nuclear localization signal (NLS) or a nuclear export signal (NES), respectively. The best characterized nuclear import pathway is mediated by the importin-β1/importin-α heterodimer [31], which requires the participation of auxiliary proteins and facilitates the effective translocation of cargoes based on a RanGDP/GTP gradient between the cytoplasm and the nucleus (see references 32,33). It has been shown that the nuclear accumulation of specific importin-β1/α cargoes also requires active MT and actin cytoskeletons (see for example 34–36).

In *Aspergillus nidulans*, the nuclear localization of importin-α and importin-β1 homologues, KapA and KapB, has been partially described in the literature [26,30]. In this work we define new features and show that the function of those karyopherins is not limited to the nucleus and its vicinity. Additional cytoplasmic pools already exist, which move bi-directionally to the tip or distal regions. These anterograde and retrograde movements are simultaneous for both karyopherins and depend mainly on MTs. Consequently, mutations in *nudA* or *nudK*, affecting the dynein motor complex, inhibit KapA cytoplasmic transport. Similarly, the deletion of the *A. nidulans* kinesin-3 coding gene, *uncA*, affects both nuclear and cytoplasmic localizations of the main nuclear import complex. Common features between this transport mechanism and injury signaling in neurons are discussed. Overall, these results link processes that occur at different cellular locations, such as polar growth and environmental signaling at the tip, cargo trafficking through cytoplasmic filaments along the length of the cell and nuclear transport through the NE.

## Results

### *Aspergillus nidulans* contains soluble and non-soluble pools of nuclear import machinery components KapB and KapA

In the first systematic characterization of the nuclear transport machinery in a filamentous fungus, fluorescent tagging and deletion analyses permitted our group to define a general karyopherin distribution map during the cell-cycle [26]. Nuclear transporters were defined as the “soluble fraction” of the nucleo-cytoplasmic trafficking machinery. This definition refers to the transient relationship with the NPC, considered as a "static" structure [37]. The coenocytic cell organization for *A. nidulans* as for other filamentous fungi might impose restrictions or variations to this concept for nuclear transporters. With the aim to discover and study additional features for specific karyopherins we used as a first step a cell-fractionation procedure to discriminate between possible organelle-associations or formation of complexes.

**Figure 1:**
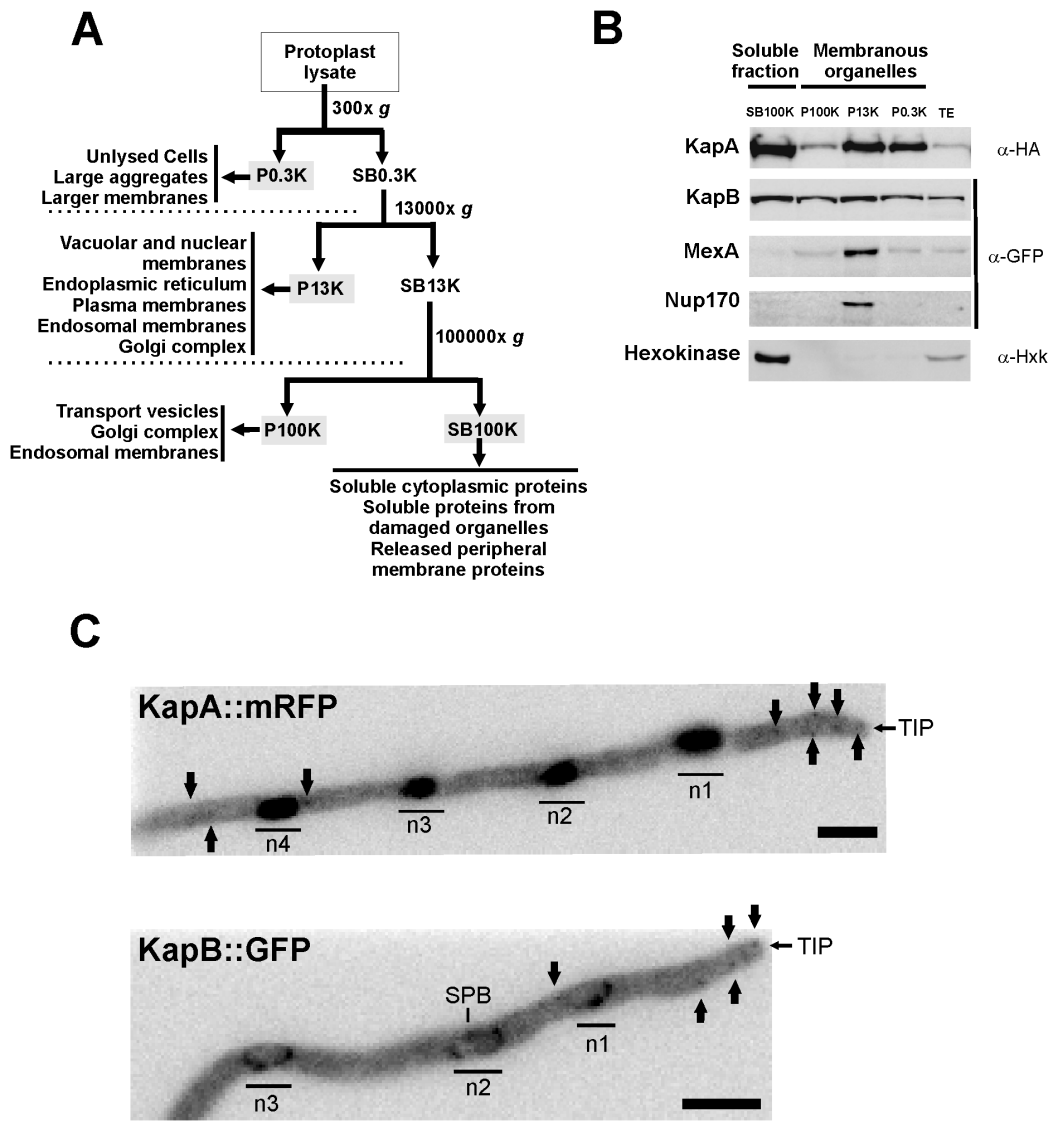
Subcellular pools of karyopherins KapA and KapB. A) Fractionation procedure for strains expressing KapB::GFP or KapA::mRFP (based on reference [38]). The diagram shows the predicted content of each fraction generated. B) Western-blot experiments showing the karyopherin-α or -β1 content of each cellular fraction. Antibodies used in each case are indicated on the right. The perinuclear RNA-export factor MexA, the NPC-core nucleoporin Nup170 and hexokinase (Hxk), the latter as a marker for cytoplasmic proteins, were used as controls. C) Maximum projection fluorescence microscopy images of KapA::mRFP (top) and KapB::GFP (bottom). KapA::mRFP accumulates mainly in nuclei and small cytoplasmic patches (black arrows) located at the tip as well as subapical and distal regions. KapB::GFP locates at the nuclear envelope. Small cytoplasmic patches (black arrows) can be observed at the tip, subapical and distal regions. Scale bar = 5 µm.

Following the protocol developed by Rodríguez-Galán and coworkers [38], protoplasts from strains under study were obtained, then mechanically lysed and subsequently divided by different centrifugation steps into four fractions: P0.3K, P13K, P100K and SB100K (see Experimental Procedures and Figure 1A; TE stands for total extract obtained after direct lysis of protoplasts). The SB100K fraction constitutes the cytoplasmic soluble content and proteins which are detected in the other three fractions are derived from membranous organelles, large aggregates or attached to membranes. We used three standards to verify that fractionation was adequately and effectively performed (Figure 1B). Firstly, we followed the fractionation of the GFP-tagged NPC-core nucleoporin Nup170 as a nuclear membrane marker that should be detected exclusively in the NE-containing fraction (Figure 1B; [24,39]). Secondly, we tracked GFP-tagged MexA, a RNA export factor, which also exhibits a perinuclear localization [26]. Finally, hexokinase (Hxk) was used as an exclusive marker of the cytoplasmic soluble fraction [38]. As expected, Nup170 and MexA were detected mainly or exclusively to the P13K fraction, containing nuclear membranes, while Hxk was detected only in the SB100K cytoplasmic soluble fraction (Figure 1B).

Once the method was validated with our internal controls, we analyzed the distribution pattern of HA-tagged KapA and GFP-tagged KapB (Figure 1B). The results showed that, in contrast with previous fluorescence studies [26], all subcellular fractions, either membrane-associated or soluble, contained KapA or KapB pools. A deeper analysis of the fluorescence of haploid strains expressing KapB::GFP or KapA::mRFP, allowed us to visualize not only the main nuclear pools but also additional accumulations in the cytoplasm (Figure 1C). Small spots or accretions of KapA and KapB were detected at distal and subapical regions (black arrows), but also at the tip. These results led us to study these additional KapA and KapB subpopulations, and elucidate the general features of their transport mechanisms.

### Cytoplasmic Pools of importins-β1 and -α Move Bidirectionally

**Figure 2:**
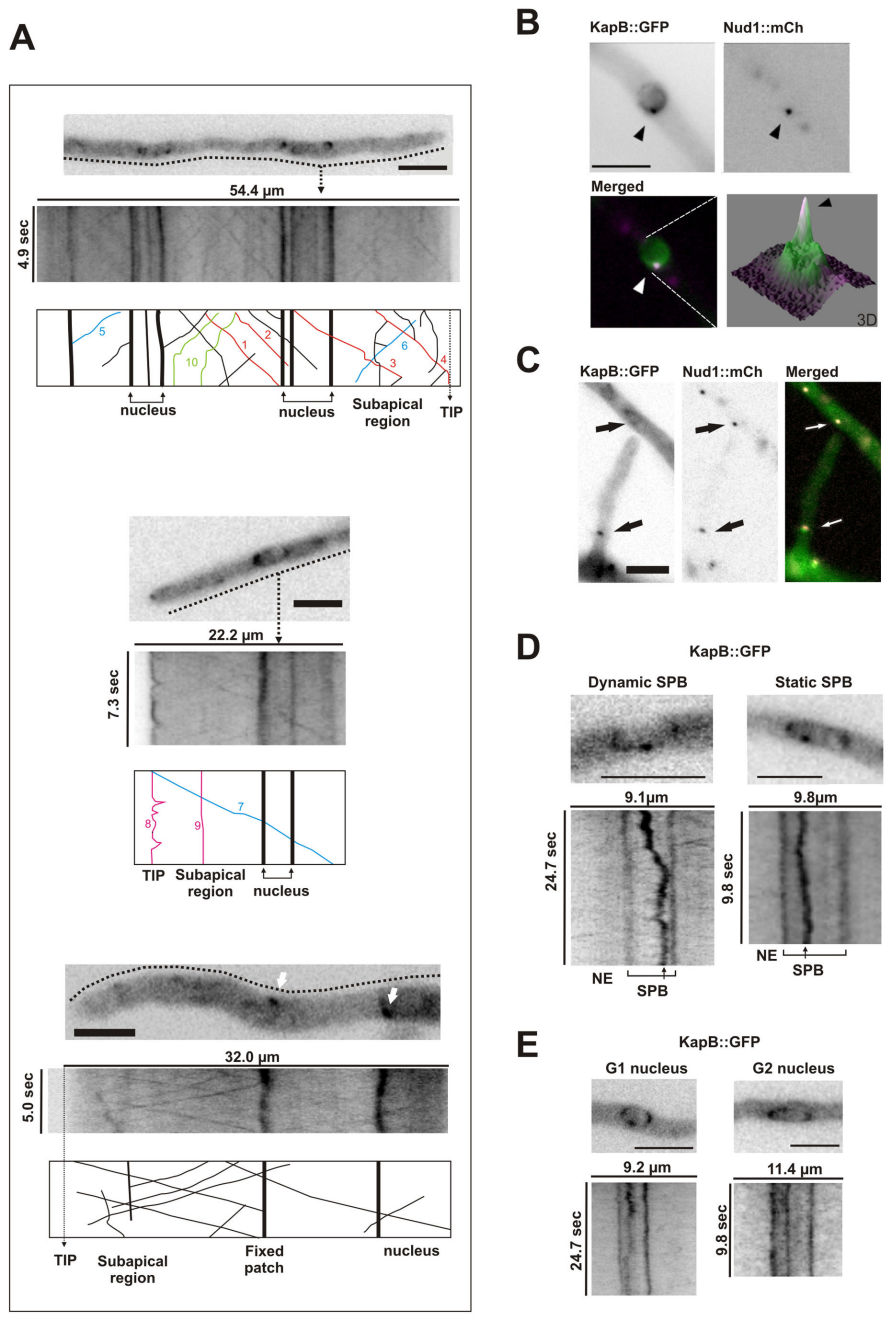
Localization and dynamics of KapB::GFP in vegetative hyphae. A) Movement of KapB::GFP patches through the cytoplasm of vegetative hyphae. The insets correspond to three video streams (the lower inset corresponds to Video S1). Each microscopy image is the first of a time stack with an associated kymograph shown below, representing the region indicated with a dotted line which covered the complete width of the hyphal tube. Diagrams at the bottom of the kymographs are included for better understanding of the trajectories followed for detected patches. Colors indicate the following: blue, retrograde movement of KapB::GFP patches; red, anterograde movement; green, motile patches that suddenly stop; and magenta, static patches. The lower inset was included to show fixed non-motile cytoplasmic patches of KapB::GFP. B) Co-localization studies of KapB::GFP with Nud1::mCh, the latter used as a marker of SPBs [26,40]. Bottom left, a 3D diagram of fluorescence intensity, with a clearly visible peak corresponding to the SPB (black arrowhead). C) KapB locates to cytoplasmic MTOCs. Arrows indicate KapB::GFP co-localization with Nud1::mCh at the cytoplasm. D) Dynamics of the SPB, observed through KapB::GFP. Left, a SPB displacing through the nuclear envelope. Right, an immobile SPB. D) KapB::GFP distribution at the nuclear envelope both at G1 and G2 phases. At G2, KapB::GFP accumulated mainly at NE regions closest to and farthest from the tip. Kymographs of the corresponding stream acquisitions are shown in the lower panels. For all images, scale bar = 5 µm.

We centered firstly on KapB because it is predictably the true transporter of the import complex, while KapA acts as the cargo adapter [32]. We acquired video streams from cells of a strain expressing the KapB::GFP fusion (MAD1266) to determine whether those spots described in Figure 1C were mobile and, if that was the case, describe the main features of this movement (Figure 2A; Video S1, which corresponds to the lower kymograph). KapB::GFP patches moved from distal regions towards the tip (anterograde movement, see trajectories in red for patches 1, 2, 3 and 4, Figure 2A) and, inversely, from the tip to distal regions of apical compartment (retrograde; in blue patches 5, 6 and 7 in diagrams from Figure 2A). Kymographs illustrate this bidirectional motility and show that multiple fluorescent patches followed common trajectories to reach the tip and distal regions (parallel lines in kymographs). The average speed measured for KapB::GFP patches at 37°C was 2.56 ± 0.88 μm/s in anterograde direction and 3.08 ± 0.75 μm/s in retrograde direction (n= 10 patches, different cells, in each direction). Some patches reached the dome of the tip (number 4 in diagrams of Figure 2A). Fixed patches were also detected at the tip and subapical regions of vegetative hyphae (in magenta, numbers 8 and 9 in middle inset of Figure 2A, respectively). Mobile patches that stopped suddenly were also detected (in green, two trajectories labeled with number 10 in upper inset of Figure 2A). 

In their movement, patches crossed strong KapB::GFP accumulations both at the nuclear envelope or the cytoplasm (see white arrows in Figure 2A, lower inset), which corresponded to either the spindle pole bodies (SPB) or MTOCs, as shown in co-localization studies with mCherry-tagged Nud1 (Figure 2B and 2C, respectively [40]). 

The analysis of KapB::GFP also allowed us to follow the positioning of the SPB. We observed that SPBs remained static in most of the cases but also showed a limited movement through the NE (Figure 2D). Furthermore, the distribution of KapB::GFP at the NE was different depending on the cell-cycle phase. At G1, we observed that KapB::GFP accumulated at nodes distributed through the NE. However, at G2, it was mostly detected at opposite poles of the nuclear envelope -those farthest and closest to the tip (Figure 2E).

**Figure 3:**
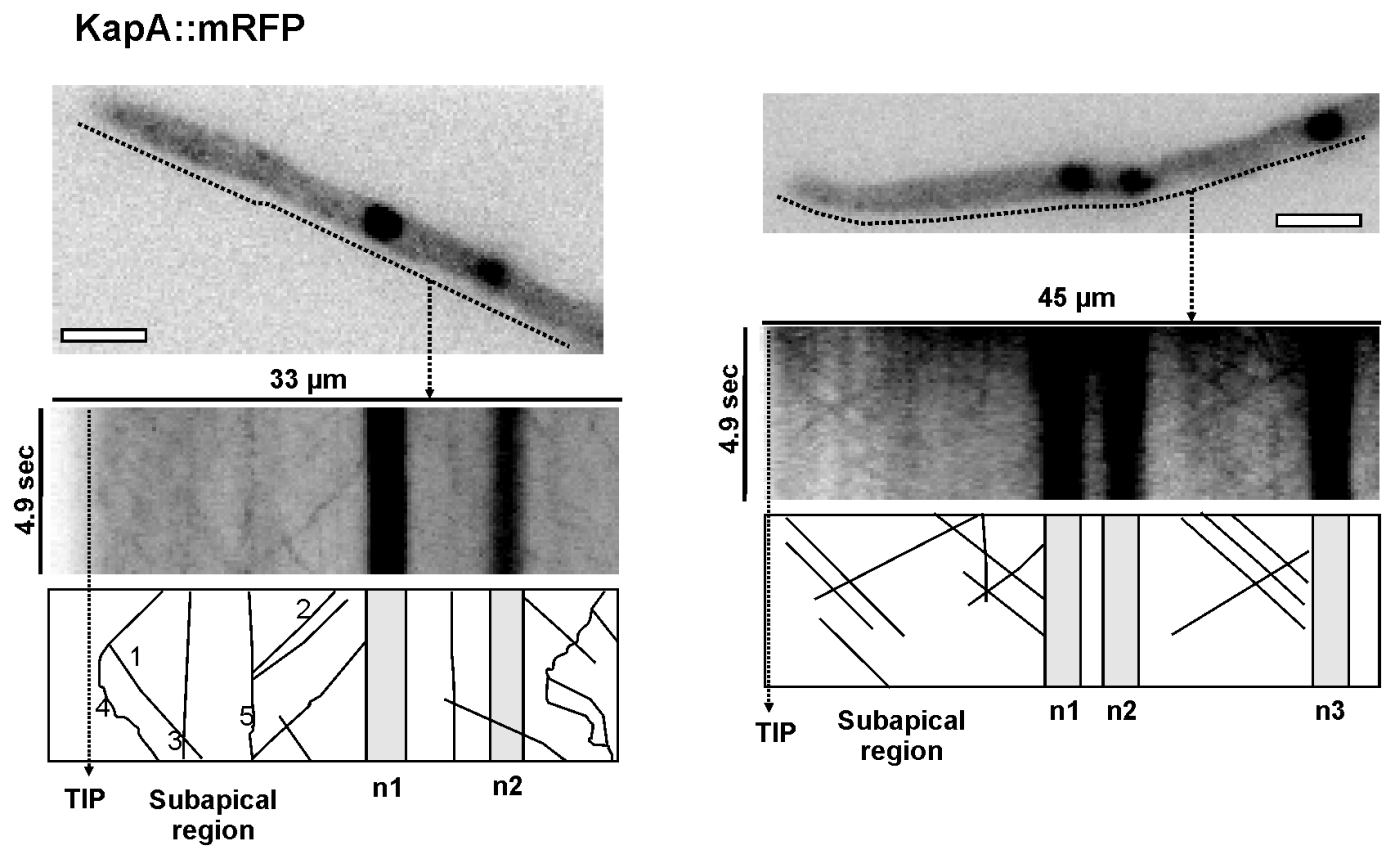
KapA::mRFP dynamics in vegetative hyphae. Movement of KapA::mRFP patches through the cytoplasm of vegetative hyphae. Kymographs illustrate the motility of KapA::mRFP along the hyphal region analyzed (dotted line). The diagrams below are included to facilitate the understanding of the kymograph. Numbers indicate: 1, retrograde movement of KapA::mRFP patches; 2, anterograde movement; 3, mobile and fixed accumulations at the subapical region; 4, entry to the tip; and 5, fixed patch. Scale bar= 5 µm. See Video S2 for the cell shown on the right side.

*Aspergillus nidulans* importin-α homolog KapA was previously described as a nucleoplasmic karyopherin [26]. However, its activity as the main, possibly the unique, cargo adapter in the importin-β1 pathway [31] and the observation of cytoplasmic spots (Figure 1C) led us to study the possible subcellular movement of this karyopherin. Of note, KapA::mRFP fluorescence intensity was extremely weak, making its detection more difficult than in the case of KapB::GFP patches. To maintain the quality of all frames in the streams acquired, we decreased both number and exposure time of each frame. With these changes in stream-capture parameters, we were able to follow KapA::mRFP movement (Figure 3; Video S2, which corresponds to the kymograph on the right). Both kymographs in Figure 3 show that KapA::mRFP patches followed specific trajectories reaching the hyphal tip and distal regions with an average speed of 3.16 ± 0.56 μm/s in anterograde direction and 3.22 ± 0.75 μm/s in retrograde direction, respectively (n= 10 patches in each direction; numbers 1, 2 and 4 in the diagram from Figure 3; Video S2). Non-mobile spots were also observed (numbers 3 and 5 in Figure 3) but they did not resemble cytoplasmic MTOCs as occurred with KapB::GFP. 

### KapB and KapA move simultaneously through the cytoplasm

The similarities described in the previous section regarding the cytoplasmic pattern of KapB::GFP and KapA::mRFP led us to investigate the possibility of a simultaneous transport. With this aim, the strains expressing either KapB::GFP or KapA::mRFP were crossed. Heterokaryons were obtained and they produced mature cleistothecia and ascospores. However, it was not possible to obtain a descendant expressing both tagged karyopherins suggesting that such genetic combination was lethal in haploid strains. Thus, we generated a diploid strain expressing both chimeras and analyzed KapB::GFP and KapA::mRFP localization simultaneously using dual channel acquisition (see Materials and Methods).

**Figure 4:**
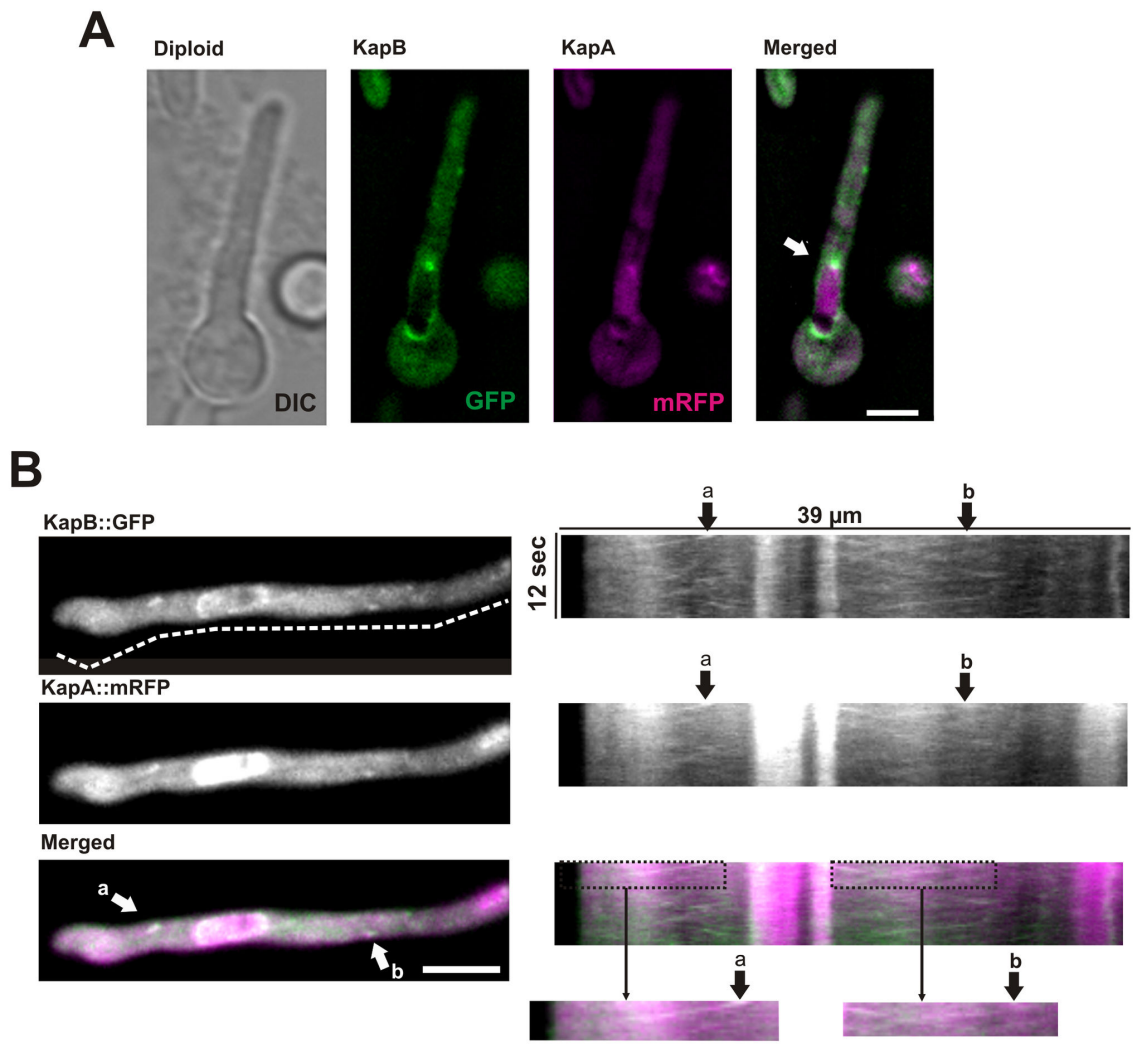
Localization and dynamics of KapB::GFP and KapA::mRFP in diploid hyphae. **A**) Co-localization (white after merging magenta, mRFP, and green, GFP, channels), of KapB::GFP and KapA::mRFP at the SPB indicated with an arrow. B) Movement of KapB::GFP and KapA::mRFP patches through the cytoplasm of diploid vegetative hyphae. Images on the left correspond to a selected frame from the stream acquisition shown in Video S3 (see Materials and Methods for simultaneous analysis of KapA and KapB movement through dual-channel acquisition). Kymographs illustrate the movement of both importins along the hyphal region analyzed. White arrows indicate two mobile patches (a and b) where KapA::mRFP and KapB::GFP co-localize. For all images, scale bar = 5 µm.

We validated the use of this diploid strain with the confirmation of KapB::GFP perinuclear and KapA::mRFP nucleoplasmic fluorescence (Figure 4A; [26]). We were also able to follow cytoplasmic patches composed of both karyopherins moving simultaneously in both anterograde and retrograde directions (Figure 4B, right block and Video S3). The speed of KapB::GFP and KapA::mRFP patches in the diploid strain was significantly lower than that described in the previous section for fusions expressed in haploids (2.26 ± 0.65 μm/s in anterograde direction and 2.15 ± 0.88 μm/s in retrograde direction; a 21-32 % reduction; n= 10 patches in each direction; p < 0.05 in both comparisons). Since we focused exclusively on simultaneous patches for speed measurements, this decrease could be a consequence of the presence of two fluorescent tags, causing a detriment in the efficiency of the complex formation or transport. Although we analyzed a representative number of simultaneous KapA and KapB patches through dual channel acquisition, we cannot discard the possibility of cytoplasmic subpopulations of either KapA::mRFP or KapB::GFP moving independently. This statement is based, on one hand, on the fact that KapA can bind proteins independently of KapB [39] and, on the other hand, on the formation in the diploid strain of KapA/KapB heterodimers in which only one or neither partner were tagged with fluorescence. Finally, fluorescence studies of KapA::mRFP and KapB::GFP in the diploid strain show that they co-localize at a specific region of the NE, which, based on co-localization studies with KapB::GFP and Nud1::mCh (Figure 2B and 2C), may coincide with the SPB (white arrow in Figure 4A).

Overall, the results hitherto shown demonstrate that vegetative hyphae contain different subpopulations of both karyopherins. These pools move simultaneously, covering long distances within the cell and reaching both the tip and distal regions. The following sections will focus on the study of the role of actin and tubulin cytoskeletons in this intracellular traffic of KapA and KapB.

### A minor pool of KapB patches may move together with RabA/Rab5-early endosomes

We analyzed whether KapB could be transported in specific subpopulations of endosomes, i.e., those defined by the Rab5 homologue RabA [16]. Several reasons led us to concentrate on a hypothetic KapB transport on RabA/Rab5 early endosomes. Firstly, RabA shows the most similar dynamics compared to that described for KapB [16]. Secondly, Rab6/RabC marks Golgi equivalents [41] while Rab7/RabS mediates fusion of late endosomes/vacuoles [42] and its dynamics is completely different to that shown by KapB. Finally, Rab4/RabF/An9072 and Rab11 have not been functionally characterized in this model fungus.

**Figure 5:**
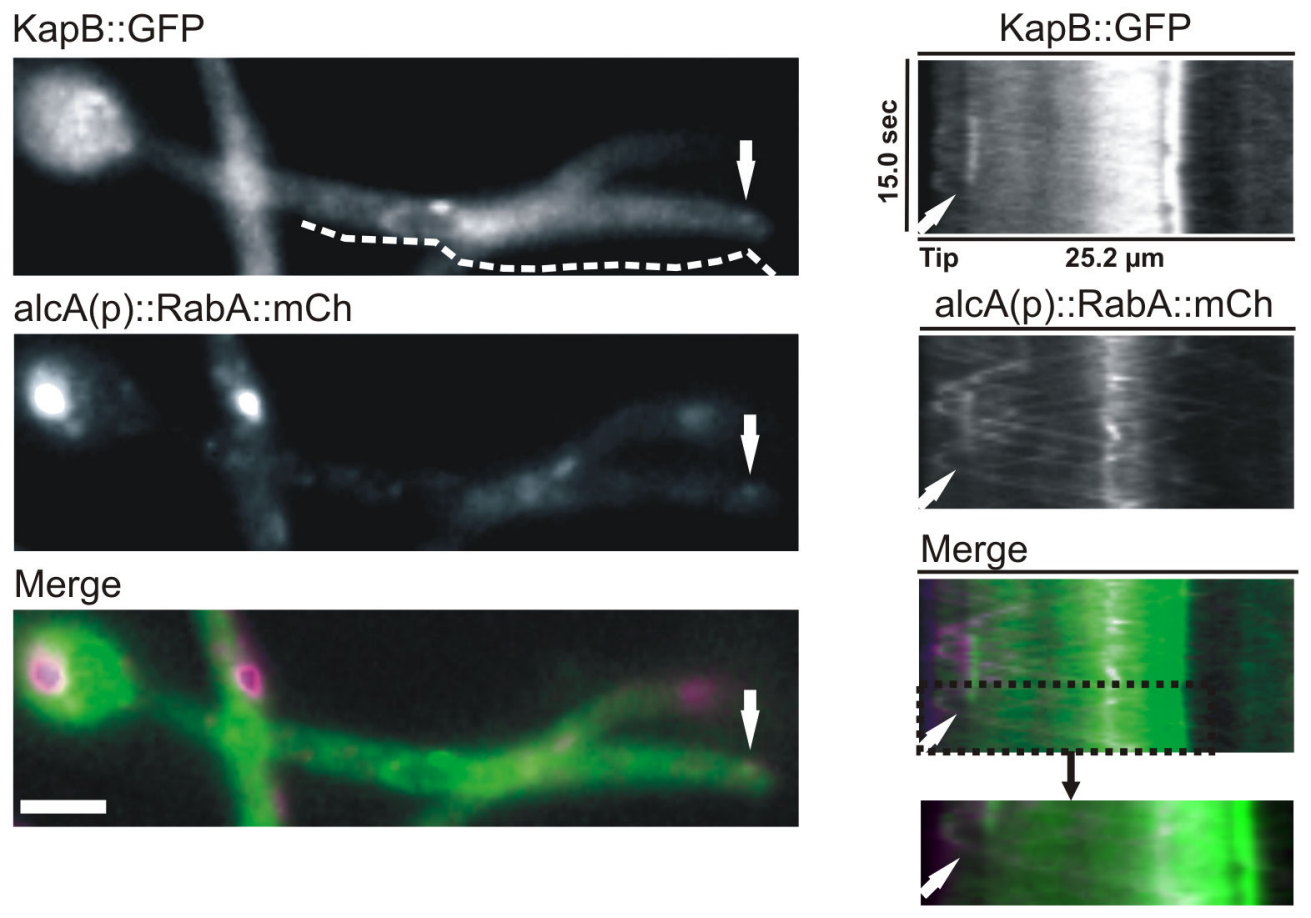
Comparison of KapB::GFP and RabA::mCh movement. The dynamics of KapB::GFP patches was compared to that of Rab5 (RabA)-labeled early endosomes. Images on the left correspond to a selected frame from the stream acquisition shown in Video S4 (see Materials and Methods for simultaneous analysis of RabA and KapB movement through dual-channel acquisition). Kymographs illustrate the movement of both proteins along the hyphal region analyzed. The white arrow indicates a mobile patch where KapB::GFP and mCh::RabA co-localize. Scale bar = 5 µm.

Consequently, we obtained a haploid strain expressing mCh-tagged RabA driven by the ethanol-inducible *alcA* promoter and KapB::GFP (see Materials and Methods; Figure 5). Our streams and kymographs showed specific patches of KapB::GFP moving simultaneously with Rab5 endosomes in vegetative hyphae (see white arrows in Figure 5; Video S4). However, we also observed multiple KapB::GFP patches not co-localizing with RabA labeled endosomes. These results suggest that a minor pool of KapB may move together with RabA-endosomes while the major importin-β population seems to move independently of this type of endosome (see Discussion).

### KapB and KapA cytoplasmic mobility depends on microtubules

The observation of KapA and KapB moving through the cytoplasm following defined trajectories and their entry to/exit from the hyphal tip is consistent with their hypothetic transport through MTs and/or actin cables. Previous works also showed interactions of Armadillo domain (ARM; [43])-containing factors (predictably, KapA contains 10 ARM domains) with both actin and tubulin cytoskeletons (see for example references 44–48). Thus, we examined the role of actin and tubulin cytoskeletons in the movement of KapA::mRFP and KapB::GFP by following their cytoplasmic localization after the addition of tubulin or actin destabilizing drugs benomyl and latrunculin B, respectively.

**Figure 6:**
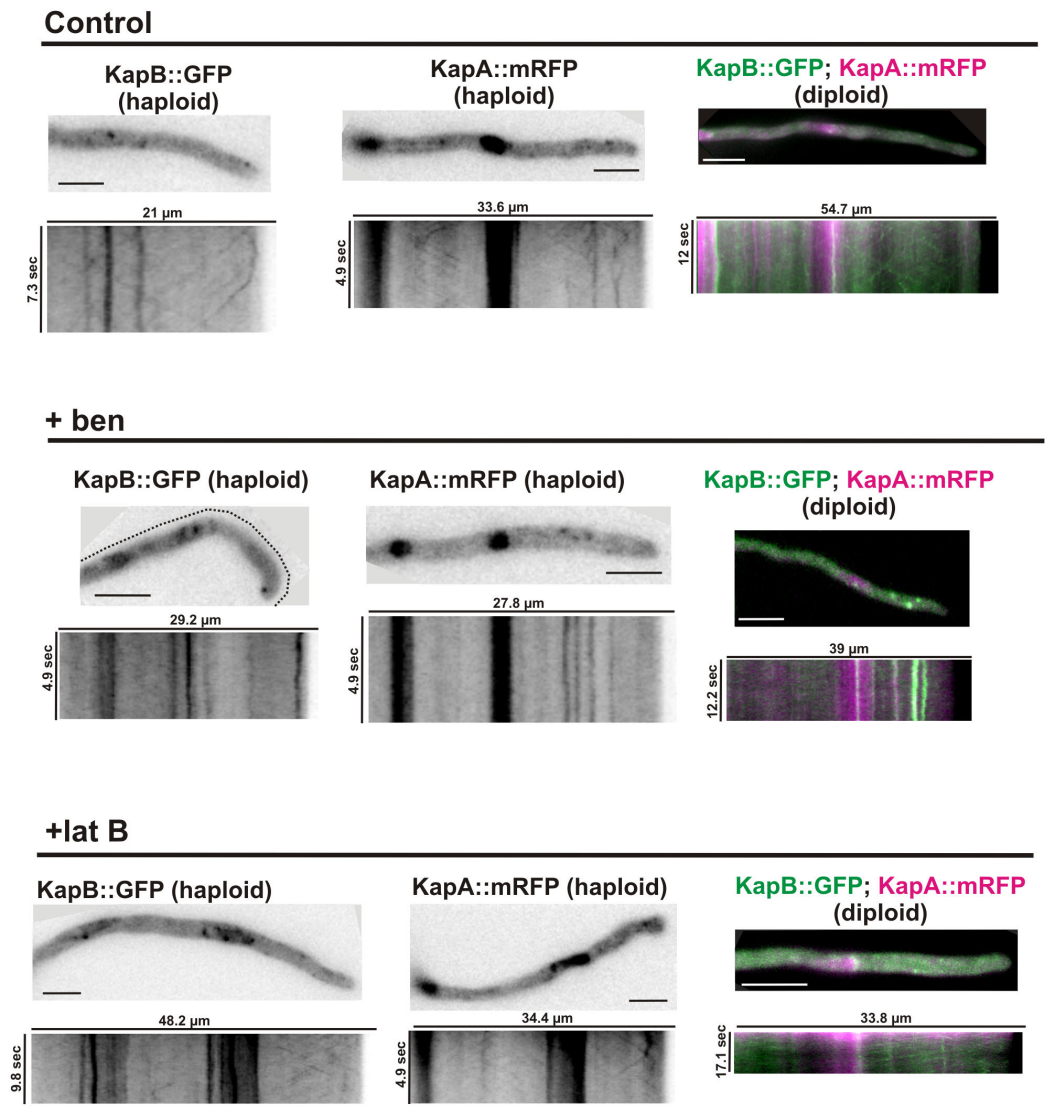
Effect of cytoskeleton destabilizing drugs on KapB::GFP and KapA::mRFP expressed in haploid or diploid hyphae. KapB::GFP and KapA::mRFP dynamics were followed in haploid strains (left and middle columns, respectively) as well as both simultaneously in a diploid strain (right column, merged image of both fluorescence channels is shown for each condition). Minimal medium (control); supplemented with 3 µg/ml benomyl (+ben); supplemented with 40 µg/ml (100 µM) Latrunculin B (+lat B).

Compared to KapA and KapB dynamics in untreated cells (Figure 6, control; see also previous sections), benomyl addition (3μg/ml) impaired the movement of both karyopherins. Motile patches were not observed in kymographs of streams taken from either haploid or diploid strains. Non-mobile accumulations were now visible along the cytoplasm but lacking specific distribution (Figure 6, +ben).

The addition of the actin-destabilizing drug latrunculin B (100µM) did not inhibit completely the movement of either KapB::GFP or KapA::mRFP cytoplasmic patches, which continued moving simultaneously in both anterograde and retrograde directions (Figure 6, +latB). However, the average speed significantly decreased to 2.18 ± 0.57 μm/s and 2.13 ± 0.63 μm/s in anterograde and retrograde directions, respectively (these are the average values calculated considering both KapB::GFP and KapA::mRFP patches; n= 20 patches in each direction, 10 corresponding to KapB::GFP and 10 to KapA::mRFP; p < 0.01 in both comparisons). This is a reduction of ~24 % (anterograde) and ~33 % (retrograde), respectively, compared to the values calculated for non-treated cells (Figure 6, control).

### Mutations in kinesin-3 and dynein motors severely affect KapB and KapA dynamics

**Figure 7:**
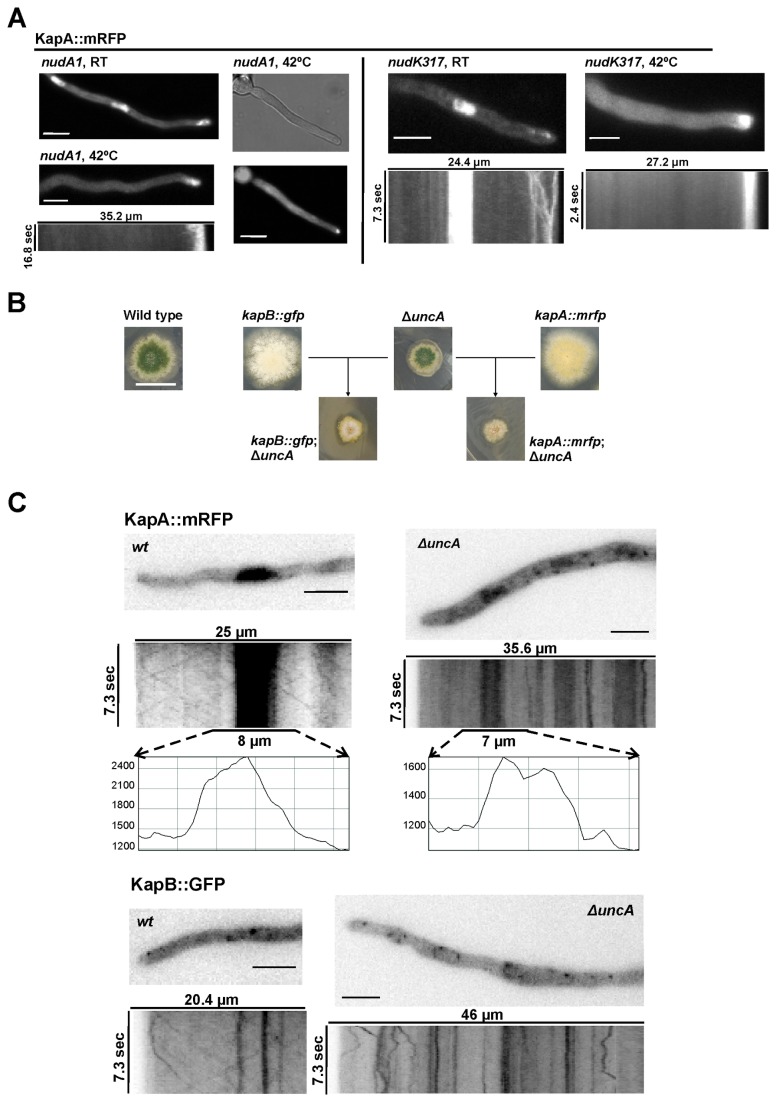
Effect of mutations affecting cytoskeleton-dependent transport on KapB::GFP and KapA::mRFP localization. A) KapA::mRFP localization in *nudA1* and *nudK317* mutant backgrounds. Strains were shifted to 42°C for 2 hours before analysis. Scale bar = 5 µm. B) Phenotype of strains expressing KapB::GFP or KapA::mRFP in wild-type and Δ*uncA* backgrounds. Scale bar = 2 cm. C) KapB::GFP and KapA::mRFP dynamics in wild type (wt; left) and Δ*uncA* hyphae (right). Frames were selected from stream acquisitions corresponding to the kymographs shown. Graphs are fluorescence intensity plots for KapA::mRFP at the regions indicated by the lines above them. Scale bar = 5 µm. See Videos S5 and S6 for Δ*uncA* mutant cells.

The dependence of KapB and KapA movement on MTs led us to study the molecular motors related to transport through this cytoskeleton. Previous work on the cellular localization of dynein or dynactin subunits showed their capability to move through the cytoplasm in patches, using MTs as tracks [49]. In addition, NudK, a component of cytoplasmic dynein/dynactin, has been shown to be an *in vivo* interactor of KapA in *Aspergillus nidulans* [39]. Thus, we analyzed a hypothetic functional requirement of the dynein/dynactin complex for the cytoplasmic movement of the cargo adapter. Strains expressing KapA::mRFP in either *nudA1* [50] or *nudK317* [51] thermo-sensitive mutant backgrounds, the former affecting the heavy chain of dynein and the latter the Arp1 subunit of dynactin, were obtained by meiotic recombination. Similar results were obtained in both backgrounds (Figure 7A). At room temperature, KapA::mRFP accumulated at the subapical region of hyphae in both genetic backgrounds while it remained visible in nuclei. At the restrictive temperature of 42°C, importin-α maintained a subapical localization but it was not accumulated in nuclei (we show a germling in Figure 7A in which no KapA::mRFP nucleoplasmic accumulation can be observed). We did not detect KapA::mRFP patches moving along the cytoplasm (see kymographs in Figure 7A). These results clearly show the requirement of the dynein/dynactin complex in KapA movement and suggest that it facilitates KapA transport and accumulation in nuclei.

It has recently been reported the existence of diverse MT populations in filamentous fungi [52]. Detyrosinated MTs (dtyrMT) would maintain the tubule structure in mitosis while tyrosinated MTs (tyrMT) would form the mitotic spindle [7,52]. The activity of *A. nidulans* kinesin-3, UncA, was linked to the intracellular transport through dtyrMTs. Thus, we analyzed a possible role for UncA in the cytoplasmic transport of KapB and KapA. We obtained strains expressing either KapB::GFP or KapA::mRFP fusions in a Δ*uncA* genetic background by two methods: firstly, by transformation of the Δ*uncA* strain SNZ9 (Table 1; [52]) with the DNA cassettes coding for each tagged fusion; and secondly, by meiotic recombination between strain SNZ9 and strains expressing either KapB::GFP or KapA::mRFP. Both transformants and descendents were phenotypically indistinguishable. The growth defect of the generated strains indicated a genetic interaction between the absence of kinesin-3 of *A. nidulans* and both tagged karyopherins (Figure 7B), suggesting a functional relationship of UncA with this nuclear import pathway.

**Table 1:** *Aspergillus nidulans* strains used in this study (all strains are *veA1*).

**Strain**	**Genotype**	**Source**
TNO2A3	*pyrG89*; Δ*nkuA::argB; argB2; pyroA4*	[84]
SNZ9	*pyrG89;*Δ*nkuA::argB; argB2;pyroA4,*Δ*uncA::pyroAAf*	[51]
WX3	*pyrG89; pyroA4; nudK317*	[52]
XX3	*pyrG89; nudA1, chaA1*	[50]
MAD1266	*pyrG89; wA3; pyroA4; kapB::gfp::pyrGAf*	[26]
MAD1312	*pyrG89; nup170::gfp::pyrGAf; wA2; pyroA4*	[24]
MAD1543	*pyrG89, yA2, pabaA1; argB2; kapA::mRFP::pyrGAf*	[26]
MAD2149	*pyrG89; pyroA4; nudK317, kapA::mRFP::pyrG*	This study
MAD2150	*pabaA1; kapA::mRFP::pyrGAf; nudA1*	This study
MAD2275	*yA2; argB2, argB::alcA(p)::mCh::rabA; pantoB100*	[16]
MAD2331	*pyrG89*; Δ*nkuA::argB; argB2; pyroA4*; *mexA::gfp::pyrGAf*	[26]
MAD2446	*pyrG89; wA4; inoB2; pyroA4; hhoA::mCh::pyroAAf, pacC900*	[85]
MAD2447	*pyrG89; wA4; inoB2; pyroA4; hhoA::gfp::pyrGAf, pacC900*	[26]
MAD2606	*pyrG89*; Δ*nkuA::argB; argB2; pyroA4; kapA*:: *ha*_*3x*_ *::pyrGAf*	This study
MAD2620 (diploid)	*pyrG89/pyrG89, yA+/ yA2, pabaA+/pabaA1; wA+/wA3;argB+/ argB2;pyroA+/ pyroA4; kapB+/kapB::gfp::pyrG; kapA+/kapA::mRFP::pyrGAf*	This study
MAD2621	*pyrG89; wA4; inoB2; pyroA4; hhoA::mCh::pyroAAf, pacC900; kapB::gfp::pyrGAf*	This study
MAD2622	*pyrG89; wA4; inoB2; pyroA4; hhoA::gfp::pyrGAf, pacC900; kapA::mRFP::pyrGAf*	This study
MAD2968	*pyrG89*; Δ*nkuA::argB; argB2; pyroA4*, Δ*uncA::pyroA; kapA::mrfp::pyrGAf*	This study
MAD2970	*pyrG89*; Δ*nkuA::argB; argB2; pyroA4*, Δ*uncA::pyroA; kapB::gfp::pyrGAf*	This study
BD687	*wA3;argB2, argB::alcA(p)::mCh::rabA; pantoB100; kapB::gfp::pyrGAf*	This study

At the microscopic level, we observed that kinesin-3 deletion significantly altered dynamics of both karyopherins. Nucleoplasmic accumulation of KapA::mRFP significantly decreased in the Δ*uncA* background (Figure 7C; compare inset at the left, showing wild-type background, and that at the right, Δ*uncA*). The nuclear versus cytoplasmic fluorescence intensity ratio of KapA::mRFP decreased from a mean of 2.5 ± 0.8 in the wild type background to an average of 1.5 ± 0.1 in the Δ*uncA* background (a reduction of 40 %; N = 12 nuclei in each genetic background; p = 0.00073). Kymographs, mainly the one corresponding to KapB::GFP, clearly show the erratic, short-distance displacement of cytoplasmic patches (Videos S5 and S6). No patch was detected covering long distances as observed in the wild-type background (Figure 7C, see also previous figures). These results suggest that UncA and, consequently dtyrMTs, may be required for: 1) the cytoplasmic transport of the general nuclear import complex, and 2) its recruitment to nuclear pores. However, the nature of this functional link remains to be elucidated.

## Discussion

Successful adaptation of organisms to their environment involves, on one hand, the development of particular mechanisms that confer specific advantages to compete in an ecological niche (i.e., plasmids in bacteria or secondary metabolite clusters in fungi). On the other hand, the process of evolution results in the conservation of advantageous molecular pathways that are broadly conserved among species, allowing efficient application of cellular resources to enhance growth, development, diversification and survival. This occurs, for example, with the machinery that establishes and controls cell-polarity and nuclear transport.

*S. cerevisiae* cells are uninucleated and symmetrical and become polarized in order to undergo asymmetric cell division, a process known as “budding” (reviewed by [4]). Thus, yeast cells display polarity only during specific stages of their life cycle [1]. Recent developments in elucidating the roles of the cytoskeleton during polarity establishment have been reported [1,53,54] but, in addition to the cytoskeleton, the nuclear transport machinery is relevant, since it plays a key role in the correct nucleocytoplasmic transport of asymmetrically distributed mRNAs [55]. However, signal transition between the cytoplasm and the nucleus in yeast seems less complex than in other cell types.

Synapse-to-nucleus communication in neurons requires protein messengers to be retrogradely transported hundreds of microns (even centimeters) away [56]. Neurons are constantly polarized and multiple mechanisms exist to convey synaptic signals to the nucleus (see within [56]). The importin-β1/α complex plays key roles in some of these mechanisms (see below). Multinucleated cells (hyphae) of *Aspergillus nidulans* are, like neurons, constantly polarized but in this case the nucleocytoplasmic transport of proteins must overcome long inter-nuclear distances. Thus, additional regulatory mechanisms might be required to guarantee the proper distribution of nuclear proteins. In this context, we have shown that karyopherins KapA and KapB can move long distances through the cytoplasm reaching the hyphal tip and distal regions. This is a new finding for karyopherins in fungi. This process links nuclear transport, the cytoskeleton and morphogenetic processes guided at the Spitzenkörper, the apical signaling hub that enables polar growth and co-ordinates developmental transitions (52). Furthermore, we strongly believe that although neurons are uninucleated and vegetative hyphae are multinucleated, several similarities can be drawn between synapse-to-nucleus and a putative tip-to-nucleus communication.

In neurons endocytosis of receptor-ligand complexes from axonal synapses into early endosomes has been described to be a major mechanism of signal transmission to the nucleus [57]. Endosomes are subsequently actively transported to the soma along MTs associated with dynein motors [58,59]. Another long-distance signaling mechanism with potentially critical roles in adult neurons is retrograde injury signaling from axonal lesion sites, which does not necessarily require endosomes [60]. This mechanism is based on direct interactions of cargo proteins with importin nuclear transport factors in complexes with molecular motors [61]. Basically importin-αs are found in axons in constitutive association with the retrograde motor dynein. mRNA for importin-β1 protein is found in sensory axons and is locally translated at the injury site after lesion [61]. This leads to the formation of dynein-bound importin-β1/α heterodimers, which actively transport signaling cargos to the nucleus [56,60].

Similarly, our work shows that KapA and KapB move retrogradely from the tip to distal regions. Since this movement is simultaneous for both karyopherins, it could be suggested that they move in association, although we could not demonstrate a direct interaction by using the split-YFP technique (not shown). However, in *A. nidulans* KapA and KapB also move simultaneously in the anterograde direction, suggesting that the association between the two proteins is not formed after the local translation of *kapB* mRNA at the tip in response to the signal reception, as occurs in neurons. The average speed of KapA and KapB is in the same range (μm/s) of that measured for early endosomes (EEs; [16]) and our co-localization studies suggested an EE-dependent transport of karyopherins. However, the presence of KapB mobile patches that do not co-localize with RabA opens the possibility of an alternative to EE-dependent transport, which is further supported by the direct interaction of KapA with the actin-related protein NudK, a component of the dynactin complex [39,62]. The dynein/dynactin complex is essential for patch motility and the nuclear accumulation of KapA while UncA (kinesin-3) is involved in the latter transport mechanism. Overall, it could be suggested that cytoplasmic dynein, dtyrMTs and kinesin-3 may facilitate the recruitment of this and other importins to the proximities of NPCs as gates of nuclei. Cooperation between these cytoskeleton and motor elements was previously described in fungi for the transport of EEs [19,63]. 

Latrunculin B addition slightly reduced the average pace of KapA and KapB patches. Thus, we cannot discard a minor role for the actin cytoskeleton in their transport. Mechanistic relationships between the nuclear transport machinery and actin microfilaments have been described in other organisms, as for example, the interaction between importin-α and yeast ARP2/3 complex [64] and the relationship of importins with endocytosis in metazoans [65].

The cytoplasmic movement of importins may be directed to the transport of transcription factors (TF) from other cell compartments. TFs are adapted to take advantage of nucleocytoplasmic transport mechanisms [66]. Proteomic studies revealed that more than 150 proteins contained *bona fide* NLSs at the postsynaptic density [67,68] while other analyses described that 39 TFs from the postsynaptic density are implicated in the sensory neuron response to nerve injury [69,70]. Jacob, NF-ΚB or CREB2, are only some examples of synapto-nuclear TFs (see references within [56]). However, in *A. nidulans* there is only one TF known to be located at the polarity region (the tip). The bZIP-type TF FlbB transports signals associated with environmental changes from tip to nuclei and, in consequence, activates or represses development [71,72]. The import mechanism of FlbB remains unknown, but other eukaryotes could serve as models. For example, *A. thaliana* and *Mus musculus* contain various TFs (a large number of them are bZIPs) tethered to the membrane of the endoplasmic reticulum (ER), which are imported after their proteolytic cleavage in response to specific signals (see references within [73,74]). The NLS within the cytosolic domain of ERj1p (DnaJc1) mediates, after cleavage, binding with Importin-β1 and import into the nucleus [74]. SREBP-1 (Sterol Regulatory Element-binding Protein) and SREBP-2 are two bZIP-type regulators of cholesterol metabolism which normally reside in the membrane of the ER and Golgi apparatus [75,76]. After proteolysis, they enter the nucleus through a direct interaction of the leucine zipper domain with Importin-β1 [75]. The Notch family of proteins is important for the regulation of differentiation, proliferation and apoptotic programs in vertebrates and invertebrates [77]. Notch proteins act as surface receptors and regulators of gene expression. It has been recently shown that, after the proteolytic release of the notch intracellular domain, it is imported by the importin-β1/α pathway [78].

Overall, it can be concluded that karyopherin activity in eukaryotes is not exclusively limited to the nuclear periphery. The specific features that exhibit the cytoplasmic movement of KapA and KapB in vegetative hyphae of *A. nidulans* allow us to suggest that they travel in association through the cytoskeleton to bind and subsequently import cargoes to all or specific nuclei of the syncytium. Future work will be dedicated to identifying cargoes which are differentially located in the cell and subjected to this nuclear import pathway. This research line will provide additional information on the molecular mechanisms governing this essential transport pathway in eukaryotes.

## Materials and Methods

### Strains, oligonucleotides and culture conditions

*Aspergillus nidulans* strains used in this study are listed in Table 1. Oligonucleotides used were described in [26]. Strains were cultivated in adequately supplemented *Aspergillus* minimal medium, MMA [79]. Strains MAD2621 and MAD2622 were obtained by meiotic crosses of strains MAD2446 and MAD1266 or MAD2447 and MAD1543, respectively. MAD1543 was also crossed with *nudK317* and *nudA1* mutant strains (provided by V. Efimov) to obtain strains MAD2149 and MAD2150, respectively. Strain BD687 was obtained from the cross between MAD1266 and MAD2275. Strains MAD2968 and MAD2970 were obtained from the crosses between SNZ9 [52] and MAD1543 or MAD1266, respectively.

Diploid strain MAD2620 (expressing KapB::GFP and KapA::mRFP) was obtained by culturing in selective plates mixes of protoplasts of haploid strains expressing the single fusions. The genomic cassettes bearing *kapA::mrfp*, *kapB::gfp* or *kapA::3ha* constructs were obtained by fusion PCR [80] and transformed into appropriate recipient strains.

KapB and KapA cellular localization during vegetative growth was analyzed by inoculating conidiospore suspensions in 8-well plates (Ibidi, Germani; Cat. No. 80821) containing 300 µl/well of adequately supplemented Watch Minimal Medium (WMM; [81]). The analyses of KapB and KapA localization in benomyl (3 µg/ml) or latrunculin B (100 µM) containing media were done as described by 82.

### Measurement of the Speed of KapB::GFP or KapA::mRFP Patches

The speed of the cytoplasmic patches of both KapB::GFP and KapA::mRFP (in µm/s) was calculated by dividing the length of the trajectory (µm) covered by a specific patch with the time interval. Given values are means of 10-15 measurements plus s.e.m. Statistical significance of differences observed in the mean pace of cytoplasmic patches was assessed using the *t*-test (two-tailed). Prior to this, a *F*-test for estimating unequal variances in the populations was carried out. This procedure was followed in two cases: 1) When comparing the speed values of KapA/KapB patches in the diploid with those measured in haploid strains; and 2) When comparing the speed of KapB::GFP and Kapa::mRFP patches in the presence or absence of Latrunculin B.

### Cellular fractionations

Cellular fractions were obtained following a procedure described in reference [38]. Essentially, protoplasts of each strain were obtained [83] and lysed in 0.2 M sorbitol, 50 mM potassium acetate, 2 mM EDTA, 20 mM HEPES pH 7.2 and protease inhibitor mixture from Roche Applied Science, using a Dounce homogenizer. Subsequent centrifugations of supernatants at 300, 13000 and 100000 x g generated P0.3K, P13K and P100K solid and SB100K liquid fractions. P13K, P100K and SB100K fractions were resuspended in the lysis buffer described before, precipitated in trichloroacetic acid and resuspended again in standard urea/2-mercapto-ethanol SDS-PAGE loading buffer. Equivalent samples of the different fractions were analyzed by Western blotting.

### Western-blot

Protein fractions were resolved in 10% SDS-polyacrylamide gels, electrotransferred onto nitrocellulose filters and exposed to rat anti-HA (Roche; 1/1,000), rabbit anti-hxk (Chemicon; 1/80,000) or mouse anti-GFP (Roche; 1/5,000) monoclonal antibody cocktails. Peroxidase conjugated anti-rat (Southern Biotech; 1/4,000), anti-rabbit (Sigma; 1/10,000) or anti-mouse (Jackson ImmunoResearch; 1/4,000) IgG immunoglobin were used as secondary antibodies. Peroxidase activity was detected with SuperSignal® West Pico Chemiluminiscent Substrate (Thermo Scientific).

### Light and fluorescence microscopy

Microscopic analyses were performed as described by 72] and [82. Strictly simultaneous imaging of GFP and mCherry was carried out using a Dual-View imaging system (Photometrics, Tucson, AZ), using the recommended filter set [41]. Kymographs and maximal intensity projections were made using Metamorph® software (Molecular Devices, USA).

### Isolation and manipulation of nucleic acids

The isolation and manipulation of DNA samples as well as Southern-blot experiments were performed as described in [72] and [82]. 

## Supporting Information

Video S1 **KapB::GFP movement through the cytoplasm of vegetative hyphae (Figure 2).** Videos were constructed using MetaMorph® and/or ImageJ software (7 fs, frames per second). Time scale is indicated in sec. Note the movement of KapB::GFP patches through the cytoplasm to the tip and distal regions. Patches crossed SPBs and cytoplasmic MTOCs.
(AVI)

Video S2 **KapA::mRFP movement through the cytoplasm of vegetative hyphae (Figure 3).** Video displays 7 fs and time scale is in sec. KapA::mRFP moves to the tip and distal regions in patches.
(AVI)

Video S3 **Movement of KapB::GFP and KapA::mRFP patches through the cytoplasm of diploid vegetative hyphae (Figure 4).** Both fusions move simultaneously. KapB::GFP and KapA::mRFP analysis was done using a dual-channel acquisition device (see Materials and Methods). The video combines three streams: green/GFP (up) and red/mRFP (middle) channels with a third merged video in magenta (bottom). Video displays 5 fs and time is indicated in sec.
(AVI)

Video S4 **Comparison of KapB::GFP with RabA(Rab5)::mRFP labeled early endosomes (Figure 5).** Video displays 7 fs and time is indicated in sec. The upper video shows the green channel (KapB::GFP) and the middle video the red channel (RabA::mCh). The lower is the merged video in magenta.
(AVI)

Video S5 **KapA::mRFP dynamics in Δ*uncA* hyphae (Figure 7).** Deletion of *A. nidulans* kinesin-3-coding gene, *uncA*, affects KapA::mRFP nuclear localization and inhibits the cytoplasmic movement. The video displays 7 fs and time is indicated in sec.
(AVI)

Video S6 **KapB::GFP dynamics in Δ*uncA* hyphae (Figure 7).** Both NE-associated localization and cytoplasmic movement of KapB::GFP are also affected by *uncA* deletion. Video displays 7 fs and time is indicated in sec.
(AVI)
